# Treatment of Ovarian Hyperstimulation Syndrome in a Mouse Model by Cannabidiol, an Angiogenesis Pathway Inhibitor

**DOI:** 10.1155/2022/1111777

**Published:** 2022-12-21

**Authors:** Kobra Tahermanesh, Sahar Hakimpour, Azam Govahi, Fariborz Keyhanfar, Abolfazl Mehdizadeh Kashi, Shahla Chaichian, Roya Shahriyaripour, Marziyeh Ajdary

**Affiliations:** ^1^Endometriosis Research Center, Iran University of Medical Sciences, Tehran, Iran; ^2^Department of Physiology, School of Veterinary Medicine, Shiraz University, Shiraz, Iran; ^3^Department of Pharmacology, School of Medicine, Iran University of Medical Sciences, Tehran, Iran

## Abstract

Studies suggest that ovarian hyperstimulation syndrome (OHSS) can be treated by reducing the level of vascular endothelial growth factor (VEGF). However, due to the side effects of commercially available VEGF-reducing drugs, they can be ruled out as a suitable treatment for OHSS; therefore, researchers are looking for new medications to treat OHSS. This study is aimed at investigating the effects of cannabidiol (CBD) in an OHSS model and to evaluate its efficacy in modulating the angiogenesis pathway and *VEGF* gene expression. For this purpose, 32 female mice were randomly divided into four groups (eight mice per group): control group, group 2 with OHSS induction, group 3 receiving 32 nmol of dimethyl sulfoxide after OHSS induction, and group 4 receiving 30 mg/kg of CBD after OHSS induction. The animals' body weight, ovarian weight, vascular permeability (VP), and ovarian follicle count were measured, and the levels of *VEGF* gene and protein expression in the peritoneal fluid were assessed. Based on the results, CBD decreased the body and ovarian weights, VP, and corpus luteum number compared to the OHSS group (*p* < 0.05). The peritoneal *VEGF* gene and protein expression levels reduced in the CBD group compared to the OHSS group (*p* < 0.05). Also, CBD caused OHSS alleviation by suppressing *VEGF* expression and VP. Overall, CBD downregulated *VEGF* gene expression and improved VP in OHSS.

## 1. Introduction

Ovarian hyperstimulation syndrome (OHSS) is the most serious iatrogenic complication of controlled ovarian hyperstimulation (COH) with exogenous gonadotropins in assisted reproductive techniques (ARTs) [1, 2]. In OHSS, ovarian enlargement occurs, and protein-rich fluid extravasation is induced from the vascular space toward the peritoneal cavity. A high amount of the fluid causes abdominal distention, weight gain, and intravascular emptying. In severe OHSS, this fluid is detectable in the pericardial and pleural cavities [3, 4].

The incidence of mild, moderate, and severe types of OHSS is 3-10% in all ART cycles. Nevertheless, in high-risk women, the incidence rate is estimated at 20% [4]. The pathophysiology of this condition remains unknown. Acute vascular permeability (VP), because of exposure to endogenous and/or exogenous human placental gonadotropin (hCG), is the main pathophysiological mechanism, leading to the fluid transfer from the intravascular space toward the third space [5].

Hypovolemia in the vascular system causes reduced organ perfusion, hydrothorax, electrolyte imbalance, diffuse intravascular coagulation (DIC), ascites, blood concentration, and renal and hepatic insufficiencies. Besides, venous thrombosis causes morbidity and mortality due to OHSS [6]. It is known that HCG-dependent elevation of vascular endothelial growth factor (VEGF), which can regulate VP, is the leading cause of major pathophysiological processes in OHSS. Therefore, *VEGF* targeting has been widely considered in the treatment of OHSS complications and reducing the possible clinical challenges.

Increased serum hCG levels induce *VEGF* mRNA expression in ovarian granulosa cells. Capillary and VP leaks occur in endothelial cells after *VEGF* binding to its receptor (VEGFR2) [7]. The absorption and development of large numbers of antral follicles can enhance *VEGF* formation in ART cycles. The level of *VEGF* expression is associated with some biological features of capillary leakage and blood concentration [8]. Cellular functions, such as extracellular matrix degradation, endothelial cell migration and proliferation, and morphological differentiation of endothelial cells, are involved in angiogenesis. To perform these functions and form a vessel, a precise balance between the stimulus and inhibitor signals is required. The stimulus signals include growth factors, such as VEGF, angiopoietins, integrins, and chemokines [9–11], while the inhibitory signaling molecules include interferons, thrombospondin, and other cytokines, as well as other endogenous inhibitors of angiogenesis that can directly or indirectly target endothelial cells.

The binding of cannabinoids to cannabinoid receptors, including CB2 and/or CB1 (WIN5512-2, HU210, JWH133, and THC), suppresses vascular endothelial cell survival and migration, prevents the spherical proliferation of endothelial cells, and inhibits angiogenesis [12, 13]. Cannabidiol (CBD), which is found in *Cannabis sativa,* contains about 80 cannabinoids [14]. CBD, as a nonpsychoactive compound, is used to treat neuropathic pain, cancer, and inflammation [15–17]. The effects of CBD on gynecologic disorders have been discussed in the literature. For instance, previous studies have reported the effects of CBD on pain alleviation and management of oxidative stress and inflammation in endometriosis patients [18, 19]. Evidence suggests that CBD has multidirectional properties, including anti-inflammatory, antioxidant [20], immunomodulatory, antiarthritic, neuroprotective, anticonvulsant [21], precognitive [22], antianxiety, antipsychotic, and antiproliferative [22] effects. Accordingly, it has been used to treat hypertension [23, 24], neurodegenerative diseases (e.g., multiple sclerosis, Alzheimer's disease, Huntington's disease, and Parkinson's disease), epilepsy [22, 25], neuropsychiatric disorders (e.g., anxiety disorders, depression, schizophrenia, and posttraumatic stress disorder after autism spectrum disorders) [22, 26], gastrointestinal diseases (e.g., nausea and vomiting, irritable bowel syndrome, and inflammatory bowel disease) [27], rheumatic disease [23], graft versus host disease, and cancer [25, 28]. Nonetheless, further research on most of these indications is needed to approve the clinical efficacy of CBD. So far, the antiangiogenic properties of CBD have not been fully investigated in OHSS models. Because of its highly favorable toxicological and pharmacological properties, the antiangiogenic properties of CBD were assessed in an OHSS model. The present findings indicated the antiangiogenic effects of CBD on *VEGF* gene expression in an OHSS model and supported the hypothesis that CBD can be used to treat OHSS.

## 2. Materials and Methods

### 2.1. Animals

This study was performed on 32 Syrian female NMRI mice (age, five weeks), weighing 30-35 g. They were maintained in cages in a 12 : 12 h dark/light cycle at 22 ± 2°C and had ad libitum access to food. This research was approved by the Ethics Committee of Iran University of Medical Sciences (IR.IUMS.REC.1400.278). For the experiments, female mice, aged five weeks (30-35 g), were randomly assigned to four groups (*n* = 8):

- Group 1 (control), receiving saline (0.1 mL) intraperitoneally for four consecutive days
- Group 2 (OHSS), receiving 10 IU of pregnant mare serum gonadotropin (PMSG, Folligon®-Intervet, Schering-Plow Animal Health Ltd., India) subcutaneously and 30 IU of hCG peritoneum (Chorulon®-Intervet, Schering-Plow Animal Health Ltd., Netherlands) on day 4
- Group 3 (OHSS+DMSO), receiving DMSO (32 nmol) via oral gavage two hours before PMSG injection for four consecutive days and two hours before hCG administration on the final day
- Group 4 (OHSS+CBD treatment), receiving CBD (30 mg/kg) dissolved in 1 mL of DMSO via oral gavage two hours before PMSG injection for four consecutive days and also two hours before hCG administration on the final day

A standard protocol was applied for the OHSS model [1]. All studies were carried out in accordance with the Guide for the Care and Use of Laboratory Animals (National Institute of Health Publication No. 80-23, revised in 1996) and approved by the Research and Ethics Committee of Iran University of Medical Sciences (Tehran, Iran) (IR.IUMS.REC.1400.278).

### 2.2. Permeability Assessment

The mice were anesthetized with ketamine-xylazine (Alfazyme, Ege Vet, Alfasan International BV, Netherlands). Forty-eight hours after hCG administration, weight gain and VP were assessed. VP was measured according to previous protocols [29]. Next, 0.2 mL of Evans Blue (EB) dye (5 mM, Sigma-Aldrich, USA) dissolved in distilled water was injected intravenously (IV) into the femoral vein with an insulin syringe. A saline solution (0.9% NaCl, 5 mL) was used to fill the peritoneal cavity and shaken at 21 rpm for 30 seconds after 30 minutes of waiting time. The peritoneal lavage fluid was then gently aspirated through a vascular catheter with no tissue damage. The rinsing fluids were collected for VP evaluation.

**Figure 1:**
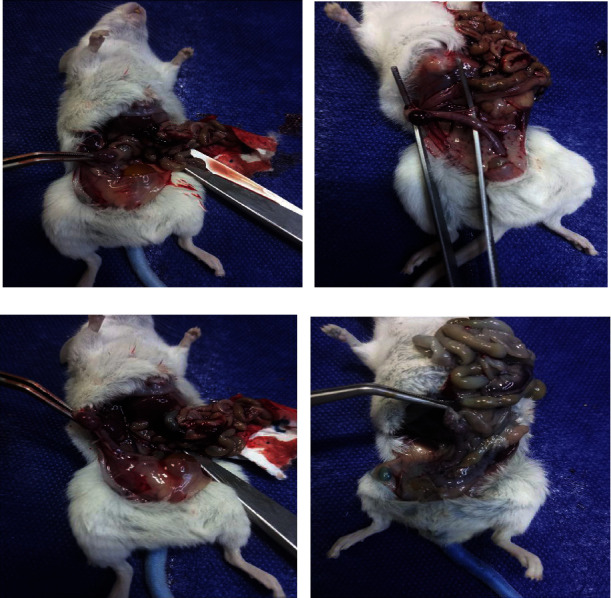
Macroscopic images of the ovaries related to the (a) control, (b) OHSS, (c) OHSS+DMSO, and (d) OHSS+CBD groups.

Following centrifugation (900 × *g*, 12 min), the EB level was calculated using a spectrophotometer at 600 nm. The concentration of dye extracted from the collected liquid is expressed as g/100 g body weight. Finally, after sacrificing the animals, their ovaries were bilaterally removed and weighed carefully. The macroscopic images of the ovaries of examined mice are presented in Figure 1.

### 2.3. Follicle Count

After the animals were sacrificed, ovarian specimens were collected and fixed in 4% paraformaldehyde (PFA) and then in a 20% sucrose solution. Next, the tissues were implanted in Tissue-Tek® (O.C.T.™ Compound; Sakura, Netherlands), followed by freezing overnight at -80°C and cutting into 6 *μ*m sections. Hematoxylin and eosin (H&E) staining was carried out using approved histological techniques. To assess the ovarian morphology, all 12 sections were mounted on a glass slide, and the number of follicles and corpus luteum was counted [30, 31].

### 2.4. Real-Time Quantitative Polymerase Chain Reaction (qPCR) Assay

RNA extraction was performed using TRIzol reagent. For this purpose, 500 *μ*L of TRIzol was added to the peritoneal fluid, and a NanoDrop spectrophotometer was used to evaluate the quality of extracted RNA. In reverse transcription (RT), a complementary mRNA strand (cDNA) was created. In this study, a SuperScript II Single-Strand cDNA Kit was used, which contains Moloney murine leukemia virus reverse transcriptase (M-MLV RT).

For cDNA synthesis from RNA, an oligo (dT) primer was used, which specifically binds to template mRNA and allows RNA transcription by the reverse transcription enzyme in the presence of dNTP. In the present study, a Thermo Fisher Scientific kit was used for cDNA synthesis. According to the kit protocol, all the following steps were performed for cDNA synthesis. Specific primers of *VEGF* and *β*-actin genes, which were designed using Gene Runner and PerlPrimer programs, were used. To confirm 100% efficiency, an online blast was also performed on the National Center for Biotechnology Information (NCBI) website (Table 1).

Real-time qPCR was performed with cDNA obtained from the peritoneal RNA deposition in each stage of the experiment. The qPCR reactions were conducted in triplicate at a final volume of 10 *μ*L, containing 250 ng of cDNA, 5 pmol of gene-specific primers, and Applied Biosystems SYBR Green Reagent, with ROX dye as a passive control dye for signal intensity. The materials were added according to the kit instructions. For real-time PCR, a primer designed for the studied gene was used; to normalize gene expression, a *β*-actin primer was employed as the internal control. The whole reaction was repeated in 40 cycles, and three different technical replicates were performed for each experimental group. In each qPCR cycle, a negative control tube was inserted, which contained sterile deionized water rather than cDNA. Moreover, the melting curve analysis determined the specificity of PCR products. All melting curves indicated one peak per product.

Additionally, the comparative CT method was employed to analyze the qPCR findings. In brief, the difference in cycle time (∆CT) was defined as the difference in the number of cycles needed for replication of the target gene and internal control (mouse *β*-actin). The ΔΔCT was determined based on the difference between the groups, and the fold change (FC) in expression was calculated using the following formula:

(1) $$\begin{array}{c} \text{FC}=2^{-∆∆\text{CT}}. \end{array}$$

The PCR efficiency of primers was 95-100% in this study.

### 2.5. Western Blotting (WB) Analysis

Considering the materials used in this study, the WB analysis was performed (25). Briefly, after removing the peritoneal fluid, it was frozen and kept at -80°C. After homogenizing the samples with a Complete Protease Inhibitor Cocktail (Roche, Germany), they were subjected to centrifugation, and protein levels were calculated using a bisynchronous acid protein (BCA) assay (Sigma-Aldrich, USA). The total protein (30 mg) was dissolved on SDS-PAGE gel (8-10%), followed by transfer to polyvinylidene fluoride (PVDF) membranes, using an electrophoretic transfer system (Bio-Rad, Germany).

Subsequently, the membranes were blocked, and incubation was performed using specific primary antibodies at 4°C overnight. The primary antibodies were loaded onto a mouse VEGF monoclonal antibody (1 : 500, Santa Cruz Biotechnology, USA) and a mouse *β*-actin monoclonal antibody (1 : 500, Santa Cruz Biotechnology, USA). The membranes were washed using 0.05% phosphate-buffered saline (PBS) with Tween 20 (PBS-T), followed by incubation with the corresponding horseradish peroxidase- (HRP-) conjugated secondary antibodies (1 : 1000) at 4°C for four hours. To detect target antigens, the blots received a substrate HRP solution (3,3-diaminobenzidine and H_2_O_2_). Following staining, the band intensities were measured using ImageJ (http://rsb.info.nih.gov/ij/) after subtracting the background and normalizing the tape density.

### 2.6. Statistical Analysis

Data analysis was performed in GraphPad Prism 8.1 (USA). Values are expressed as mean ± SD. The Kolmogorov-Smirnov test was performed to evaluate the normal distribution of data. Also, one-way analysis of variance (ANOVA), followed by Tukey's post hoc test, was conducted. A *p* value less than 0.05 was considered statistically significant.

## 3. Results

**Figure 2:**
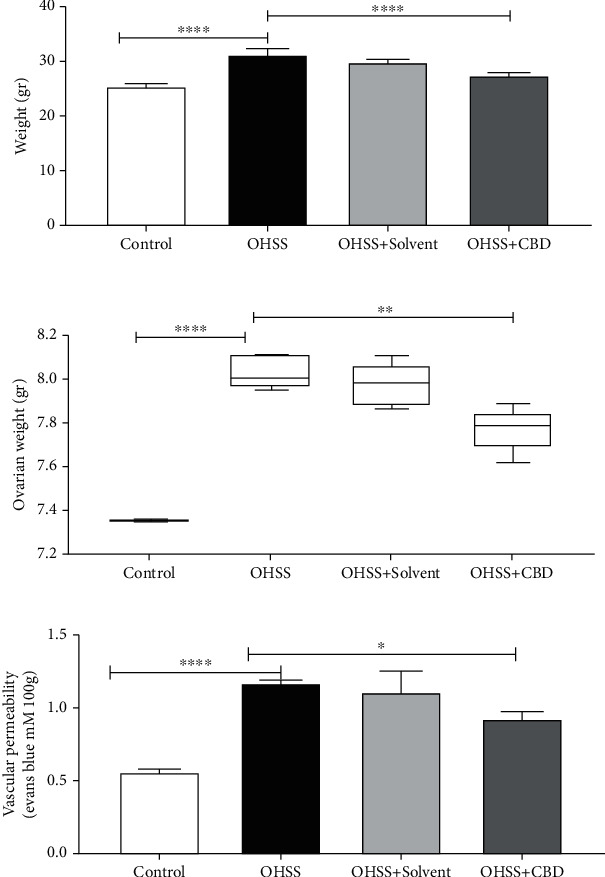
Graphical comparison of body weight (a), ovarian weight (b), and VP (c) in all groups (^∗^*p* < 0.05, ^∗∗^*p* < 0.01, and ^∗∗∗∗^*p* < 0.0001).

The body weight of the OHSS group significantly increased compared to the controls (*p* < 0.0001). The CBD group experienced a significant reduction in body weight compared to the OHSS group (*p* < 0.0001) (Figure 2(a)). The ovarian weight significantly increased in the OHSS group compared to the control group (*p* < 0.0001), while the ovaries of CBD mice weighed less than that of the OHSS group (*p* < 0.01) (Figure 2(b)). The VP significantly increased in the OHSS group compared to the control group (*p* < 0.0001), while it significantly decreased in the CBD group compared to the OHSS group (*p* < 0.05) (Figure 2(c)).

### 3.1. Measurement of Follicle Count

**Figure 3:**
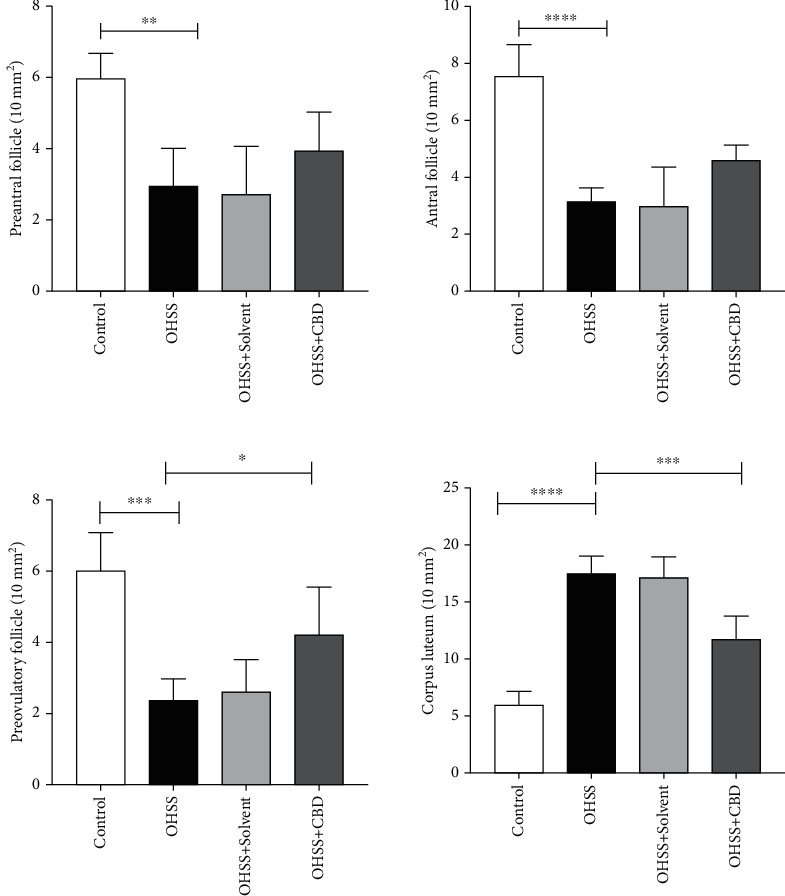
Effect of CBD on the morphology of ovary in a mice model of OHSS using quantitative morphometric analysis of the ovarian sections following H&E staining. Follicles were categorized based on the development stage presented in materials and methods. Values are presented as the number of follicles per 10 mm^2^. Data are presented as the mean ± SD (^∗∗^*p* < 0.01, ^∗∗∗^*p* < 0.001, and ^∗∗∗∗^*p* < 0.0001).

According to Figure 3, the number of antral, preantral, and preovulatory follicles was lower in the OHSS group compared to the controls (*p* < 0.05). Also, the number of preovulatory follicles was significantly lower in the CBD group compared to the OHSS group (*p* < 0.05). Based on the findings, the number of corpus luteum was higher in the OHSS group compared to the control group (*p* < 0.0001), while it was significantly lower in the CBD group compared to the OHSS group (*p* < 0.001).

### 3.2. Peritoneal Gene Expression

**Figure 4:**
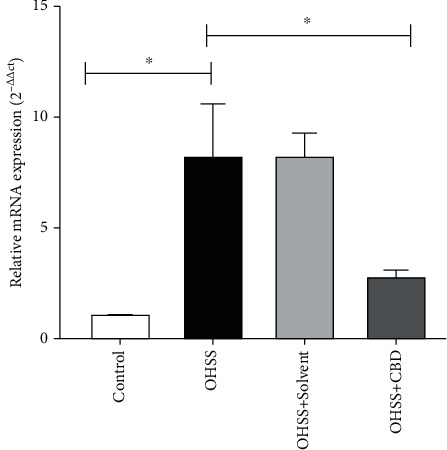
Gene expression *VEGF* in peritoneal from mice Control, DMSO, OHSS, and CBD. The mRNA levels were evaluated by qRT-PCR. Data are represented as mean ± SD (*n* = 8 for each group). *β*-Actin was applied as an internal control (^∗^*p* < 0.05).

The mean *VEGF* mRNA expression level was significantly higher in the OHSS group compared to the control group (*p* < 0.05), while the mean *VEGF* mRNA expression was significantly lower in the CBD group compared to the OHSS group (*p* < 0.05) (Figure 4).

### 3.3. Confirmation of Real-Time PCR Findings Based on the WB Analysis

**Figure 5:**
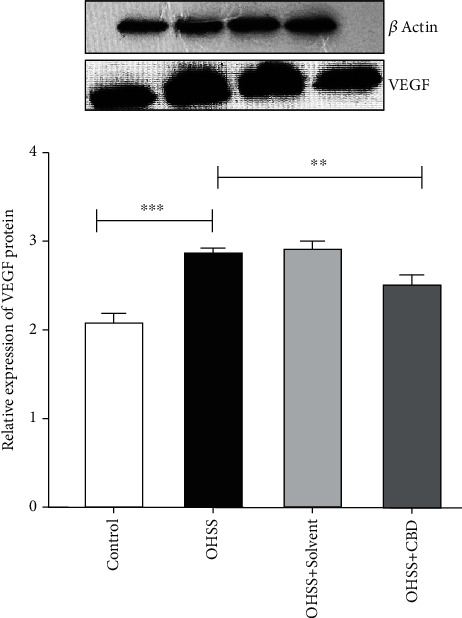
*VEGF* protein levels were assessed through Western blot. Values are the mean ± SD (*n* = 8 for each group). *β*-Actin was applied as an internal control (^∗∗∗^*p* < 0.001 and ^∗∗^*p* < 0.01).

The real-time PCR findings were approved using a WB analysis of proteins (Figure 5). The *VEGF* gene expression significantly increased in female mice in the OHSS group compared to the control group (*p* < 0.001), while it significantly reduced in the CBD group relative to the OHSS group (*p* < 0.01).

## 4. Discussion

Based on the current findings, CBD reduced body weight, VP, ovarian weight, and number of corpus luteum compared to the OHSS group. It also reduced *VEGF* gene expression in the peritoneal fluid in the OHSS model. Generally, the main known risk factors for OHSS include a history of OHSS, young age, polycystic ovarian syndrome, increased antral follicle count, high levels of antimullerian hormone (AMH), and high concentrations of hCG [32].

The management of OHSS is very important in patients undergoing in vitro fertilization (IVF) and also for physicians dealing with traumas in the emergency department. Several infertility strategies have been employed to prevent OHSS in ART cycles. In these strategies, the IVF cycles are sometimes terminated, exogenous gonadotropin is avoided, and hCG administration is delayed until the serum estradiol (E2) level drops to a safe level (coasting). Besides, the GnRH agonist is used to initiate the final egg maturation stage, albumin is injected during the oocyte retrieval, and all embryos are matured in vitro despite preventive methods. However, no clear mechanism can fully explain how these factors are involved in OHSS, although vasoactive substances released from the ovaries into the circulatory system under hCG induction are associated with this syndrome [1, 33].

The symptoms of OHSS are mainly attributed to fluid leakage from the capillaries, stimulated by an ovarian permeability agent. The formation of this fluid is increased by gonadotropic hormones, which are prescribed for fertility [34–36], and then secreted from enlarged ovaries. An increase in VP mainly results in peritoneal fluid accumulation, edema, and sometimes hypovolemia. Intravascular volume loss contributes to the complications and occasional mortality of OHSS [35]. The development of OHSS is well associated with the ovarian response to gonadotropic hormones, which involves numerous stimulated follicles and increased serum E2 levels [37, 38].

The level of vasoactive substances in the arteries or ovaries has been linked to the temporal growth of OHSS. Consequently, different substances inside and outside the ovaries contribute to OHSS, including histamine, prostaglandins, serotonin, angiotensin, prolactin, and interleukin-2 [39–41]. The present results revealed that *VEGF* is an ovarian factor, which is generated during hormonal induction and results in an increase in VP; therefore, the expression of *VEGF* gene is associated with OHSS manifestations. The increasing effect of *VEGF* gene on VP may be partly related to the effects of other vasoactive agents [42].

In vitro, exogenous VEGF is associated with nitric oxide (NO) production from target cells/organs, such as EC [42, 43]. NO increases VP by activation of protein kinase G [44]. It can bind to and disrupt cytoskeletal protein complexes [31, 32] and deplete adenosine 5′-triphosphate (ATP), leading to the dilation of tight EC bonds and rearrangement of actin cytoskeletons [45–47]. Moreover, NO modifies cytoskeletal proteins, modulates EC-tight junction (TJ) formation, and modulates the exchange of transendothelial salts. Several proteins are also associated with the formation of dynamic junctions. The association of TJ proteins (ZO-1, ZO-2, and occludin) with actin cytoskeleton [48] and cadherin-catenin complexes plays an important role [49, 50]. Disruptions in the formation of strong EC bonds are attributed to the disruption of actin cytoskeleton [47, 51] and increased *VEGF* expression [52]. In patients with OHSS, these strong intercellular connections are disrupted, and *VEGF* expression is elevated [53], resulting in the reduced level of plasminogen (SERPINE1/PAI-1), as an important factor in the extracellular matrix regeneration and angiogenesis [54].

Today, various medicines and agents have been studied to reduce *VEGF* gene expression. CBD is one of the most recent compounds used to reduce *VEGF* expression in gynecological disorders. Molecular targets, pharmacokinetics, and abuse/safety liability of CBD have been extensively discussed in the literature [55, 56]. The inhibition of *VEGF* expression in glioma cells by CBD represents its antineoplastic and antiangiogenic properties [57]. In this regard, Solinas et al. reported CBD-induced human umbilical vein endothelial cell (HUVEC) migration, in vitro sprouting and invasion, and in vivo angiogenesis in tumor cells; such effects were related to the downmodulation of many angiogenesis-related molecules [58]. This study showed that the antiangiogenic activity of CBD is effective in counteracting OHSS development. Moreover, CBD significantly suppressed two potent angiogenic agents, that is, IL-8 and CXCL16 [59].

By acting on vascular endothelial cells, CBD reduces angiogenesis and abnormalities and reduces *VEGF* gene expression in cancer cells in vivo. However, the molecular mechanism of the effects of CBD is not known yet [60, 61]. To reduce *VEGF* gene secretion, suppression of MAPK, ERK1/2, and P38 signaling pathways is required; CBD is one of the effective compounds in suppressing this pathway [62]. Although the incidence of severe OHSS is low, it remains a serious complication of IVF. Due to its pathophysiology, OHSS is managed experimentally. Many approaches have been employed to prevent OHSS. Considering the pivotal role of *VEGF* gene in the pathogenesis of OHSS, it was targeted in a previous study [63]. Decreased *VEGF* gene expression in the OHSS model reduced the pathogenesis of disease. In this study, the level of *VEGF* gene expression increased in the OHSS model, while following CBD treatment, and the expression of this gene decreased significantly in the peritoneal fluid.

## 5. Conclusion

Based on the present findings, CBD exerts potent antiangiogenic effects by influencing many associated pathways. It led to the alleviation of OHSS by suppressing *VEGF* gene expression and VP. Also, CBD downregulated *VEGF* expression and improved VP in OHSS. It is hoped that the current results can help reduce the symptoms and complications of OHSS and prevent the cancellation of IVF cycles.

## Figures and Tables

**Table 1:** List of used primers in real-time PCR.

Gene		Sequence (5′- >3′)	Length	Tm	GC%	Self-5′ complementarity	Self-3′ complementarity
Actin-beta gene (*β*-actin)	Forward primer	CAAGATCATTGCTCCTCCTG	20	58.4	50.00	6.00	1.00
	Reverse primer	ATCCACATCTGCTGGAAGG	19	57.3	52.60	6.00	0.00

Vascular endothelial growth factor (*VEGF*)	Forward primer	TGCAGATTATGCGGATCAAACC	22	59.38	45.45	5.00	2.00
	Reverse primer	TGCATTCACATTTGTTGTGCTGTAG	22	61.02	40.00	4.00	2.00

## Data Availability

The underlying data supporting the results of our study can be found in the manuscript.
